# p53/E2F7 axis promotes temozolomide chemoresistance in glioblastoma multiforme

**DOI:** 10.1186/s12885-024-12017-y

**Published:** 2024-03-07

**Authors:** Jiao Meng, Wei Qian, Zhenkun Yang, Lingli Gong, Daxing Xu, Hongbo Huang, Xinyi Jiang, Zhening Pu, Ying Yin, Jian Zou

**Affiliations:** 1 Department of Laboratory Medicine, Wuxi People’s Hospital, Wuxi Medical Center, The Affiliated Wuxi People’s Hospital of Nanjing Medical University, Nanjing Medical University, 214023 Wuxi, Jiangsu China; 2 Center of Clinical Research, Wuxi People’s Hospital, Wuxi Medical Center, The Affiliated Wuxi People’s Hospital of Nanjing Medical University, Nanjing Medical University, 214023 Wuxi, Jiangsu China; 3 NHC Key Laboratory of Nuclear Medicine, Jiangsu Key Laboratory of Molecular Nuclear Medicine, Jiangsu Institute of Nuclear Medicine, 214063 Wuxi, China; 4 Department of Clinical Laborator, Kunshan Hospital of Traditional Chinese Medicine, Kunshan, 215300 Suzhou, Jiangsu China

**Keywords:** E2F7, p53, Chemoresistance, Temozolomide, Glioblastoma multiforme

## Abstract

**Background:**

Glioblastoma multiforme (GBM) is the most aggressive form of brain cancer, and chemoresistance poses a significant challenge to the survival and prognosis of GBM. Although numerous regulatory mechanisms that contribute to chemoresistance have been identified, many questions remain unanswered. This study aims to identify the mechanism of temozolomide (TMZ) resistance in GBM.

**Methods:**

Bioinformatics and antibody-based protein detection were used to examine the expression of E2F7 in gliomas and its correlation with prognosis. Additionally, IC_50_, cell viability, colony formation, apoptosis, doxorubicin (Dox) uptake, and intracranial transplantation were used to confirm the role of E2F7 in TMZ resistance, using our established TMZ-resistance (TMZ-R) model. Western blot and ChIP experiments provided confirmation of p53-driven regulation of E2F7.

**Results:**

Elevated levels of E2F7 were detected in GBM tissue and were correlated with a poor prognosis for patients. E2F7 was found to be upregulated in TMZ-R tumors, and its high levels were linked to increased chemotherapy resistance by limiting drug uptake and decreasing DNA damage. The expression of E2F7 was also found to be regulated by the activation of p53.

**Conclusions:**

The high expression of E2F7, regulated by activated p53, confers chemoresistance to GBM cells by inhibiting drug uptake and DNA damage. These findings highlight the significant connection between sustained p53 activation and GBM chemoresistance, offering the potential for new strategies to overcome this resistance.

**Supplementary Information:**

The online version contains supplementary material available at 10.1186/s12885-024-12017-y.

## Introduction

Glioblastoma multiforme (GBM) is the most aggressive type of primary tumor in the central nervous system. Temozolomide (TMZ) is an oral alkylating agent that readily passes through the blood-brain barrier and is the standard first-line chemotherapy for the clinical treatment of GBM [1, 2]. However, approximately 50% of GBM patients eventually develop resistance to TMZ, leading to a significantly poor prognosis [3, 4]. Therefore, it is imperative to elucidate the mechanisms that contribute to TMZ resistance in GBM.

The cytotoxicity of TMZ is commonly attributed to the induction of DNA damage, particularly O^6^-methylguanine lesions, which induce DNA crosslinking and double-strand breaks, ultimately resulting in tumor cell death [5]. The development of TMZ-resistance (TMZ-R) in GBM is intricate and multifaceted. In addition to DNA repair deregulation mediated by O^6^-methylguanine-DNA methyltransferase (MGMT) alteration, the characteristics of cell cycle arrest, cellular quiescence and drug uptake reduction cannot be overlooked [6–8]. Although multiple attempts have made great progress, great challenges remain to be overcome in order to understand drug resistance in GBM.

The E2F family of transcription factors is encoded by eight genes form a core transcriptional axis crucial for coordinating cell cycle transitions. The heightened activity of E2Fs (E2F1, E2F2, E2F3), commonly found in many cancer types, often results from inactivation of the key regulator retinoblastoma protein (RB), overexpression of cyclin-dependent kinases (CDKs), or inactivation of CDK inhibitors [9]. In GBM, deregulation of E2F1 and E2F6 is associated with TMZ resistance [10, 11]. Additionally, E2F7, an atypical repressor of the E2F family has been described as a repressor of its downstream genes and has been shown to have oscillatory and controversial functions in cancers. It is worth considering that E2F7 may also be related to drug resistance.

In this study, we identify that the elevation of E2F7 is associated with malignance and poor patient survival. With the use of cells derived from a resistant model of TMZ in vivo [7], using serial implantation of MGMT-hypermethylated U87 cells, the extraction of stable, TMZ-R tumors and primary cells was achieved. The derived tumors and cells exhibited stable multidrug resistance both in vitro and in vivo. Previous research has indicated that E2F7 is a gene regulated by p53, with p53 binding directly upstream of the E2F7 transcription start site (TSS) [12]. Our findings shed new light on the mechanisms underlying chemoresistance in GBM and highlight the transcriptional regulation of E2F7 by p53. These insights may contribute to the development of novel therapeutic strategies to overcome TMZ resistance in GBM cells.

## Materials and methods

### Transcriptome sequencing and data analysis

Transcriptome sequencing was performed by YiKe Population Health Research Institute, Nanjing, China. Briefly, total RNA was extracted from the specified tissues using Trizol reagent (Thermo-Fisher Scientific, MA, USA), and its quality and quantity was assessed using by a Nano Photometer (IMPLEN, CA) and a Qubit 3.0 Fluorometer C (Thermo-Fisher Scientific), respectively. Poly(A)-tailed mRNA was captured and transcribed to double-strand cDNA, followed by sequencing on a HiSeq3000 system (Illumina, San Diego, CA) after library construction. Raw reads were acquired by CASAVA v1.8 and stored in FASTQ format. The human reference genome (GRCh37) was used to align the aforementioned reads. Statistical results in terms of “Read counts” were output through Htseq v 0.7.2 and corresponding “Fragments Per Kilobase Million (FPKM)” were calculated. Differentially expressed mRNAs were selected according to the criteria of *p* < 0.05 and|log fold change (FC)| ≥ 2. Gene Set Enrichment Analysis (GSEA) was performed using GSEA/MSigDB (Broad Institute, https://www.gsea-msigdb.org/gsea/msigdb/index.jsp). The sequencing and data analysis were performed on six samples, including three duplicates each, for the TMZ-sensitive (TMZ-S) and TMZ-R tissues with various constructs. The clinical data and matrix of public datasets Rembrandt and Gravendeel were acquired from GlioVis (http://gliovis.bioinfo.cnio.es/). Gene Ontology (GO), and Kyoto Encyclopedia of Genes and Genomes (KEGG) analysis were performed using Metascape (https://metascape.org/) based on the sequencing data from the indicated groups.

### Immunohistochemistry (IHC)

For Immunofluorescence (IF) staining of tissue arrays, an immunohistochemistry (IHC) antibody complex containing E2F7 antibodies and an Opal color fluorescent IHC kit (PerkinElmer, USA) were used, as previously described in other studies [13, 14]. The tissues were incubated with primary antibodies, followed by secondary-horseradish peroxidase (HRP) (Cell signaling, MA, USA), and Opal working solution (PerkinElmer). The slides were mounted with ProLong Gold Antifade Reagent containing 4’6-diamidino-2-phenylindole (DAPI). For the estimation of fluorescence intensities, all images were taken consistently in intensity and exposure time and analyzed using Image J software (National Institutes of Health, NIH, USA). E2F7 expression was scored semi-quantitatively based on an established immunoreactivity scoring (IRS) system [15]. Briefly, IRS covers a range of 1–12 as a product of the proportion score (1–4) and mean fluorescence intensity score (1–3). The proportion score represents the percentage of positive cells (1: < 25% of positive cells; 2: 25–50% positive cells; 3: 50–75% positive cells; 4: > 75% positive cells). The mean fluorescence intensity score represents the Integrated Density (IntDen) per unit area (1: faint; 2: medium bright, 3: high bright). The slides were concurrently checked and scored by two blinded pathologists. The mean IRS core was considered as the final IRS (Supplementary Table 1).

### Cell culture and reagents

The human GBM cell line U87 was purchased from a research institute (Chinese Academy of Sciences, Beijing, China) and was authenticated using short tandem repeat assays by the company GENEWIZ (Suzhou, China). All cells were cultured in Dulbecco’s Modified Eagle’s Medium (DMEM) (Hyclone, Los Angeles, USA) supplemented with 10% fetal bovine serum (FBS; Invitrogen, Carlsbad, CA) and 1% penicillin-streptomycin (P/S; Hyclone) in 5% CO_2_ at 37 °C. The TMZ-S and TMZ-R cells refer to primary cells derived from indicated tumor tissues previously established in our laboratory [7]. TMZ and doxorubicin (Dox) were acquired from Selleck Chemicals (Houston, TX, USA).

### Vector construction and transduction

The full-length cDNA encoding human E2F7 was amplified by RT-qPCR and verified by DNA sequencing. The E2F7-Flag lentivirus was constructed by inserting the cDNA sequence into the lentivirus vector GV640 (Genechem, Shanghai, China) with a Flag-tag. The p53-HA lentivirus was constructed by inserting the cDNA sequence into the lentivirus vector GV348 (Genechem) with an HA-tag. The small interfering RNAs (siRNA) targeting human *E2F7* were synthesized by Gene Pharma (Suzhou, China). *E2F7* siRNA transfection was performed using Lipofectamine 3000 (Thermo Fisher Scientific) according to the manufacturer’s instructions, and cells were collected for subsequent assays after 48 h of transfection. For the intracranial xenograft assay, an shRNA targeting *E2F7* was designed based on siRNA and inserted into the lentivirus vector GV112. The lentivirus was then stably infected into the indicated cells expressing the firefly luciferase gene (H7656, OBIO Technology, China). The Clustered Regularly Interspaced Short Palindromic Repeats (CRISPR/Cas9) system (Genechem) was used to establish p53 knock out (KO) cell lines. Briefly, cells were infected with Lenti-Cas9 lentivirus and screened with puromycin (Sigma-Aldrich, St Louis, MO). Single guide RNAs (sgRNAs) targeting the human *TP53* gene were designed and cloned into the GV371 vector (Genechem), and delivered into cell lines stably expressing Cas9. The pGL4.38 [luc2P/p53 RE/Hygro] (p53 RE-pGL4.38) (Promega, Madison, WI) was used to evaluate p53 transcriptional activity. The siRNA/shRNA and sgRNA sequences used in this study are listed in Supplementary Tables 2 and Table 3.

### Cell growth and colony formation assay

For cell growth assays, a total of 1000 cells were seeded into 96-well plates and monitored by Cell Counting Kit-8 (CCK8; Vazyme, Nanjing, China) at the designated time points according to the manufacturer’s protocol, with or without TMZ treatment. For cell growth assays, a total of 1000 cells were seeded into 96-well plates and monitored by Cell Counting Kit-8 (CCK8; Vazyme, Nanjing, China) at the designated time points, with or without TMZ treatment, according to the manufacturer’s protocol. The growth rate was calculated as follows: Value_(day4)_-Value_(day0)_/Value_(day0)_, and the inhibition ratio: The inhibition ratio was calculated as follows: growth rate_(DMSO)_-growth rate_(TMZ)_/growth rate_(DMSO)_. For the colony formation assays, 1000 cells were seeded into 6-well plates and maintained in complete medium for 14 days. The resulting colonies were fixed with 4% paraformaldehyde (PFA) (Sangon, Shanghai, China) and stained with 0.1% crystal violet (Sangon). The number of colonies were counted using an inverted microscope and analyzed with Image J software. The concentration of TMZ used for both the cell proliferation and colony formation experiments was 200 µM.

### Cell apoptosis analysis

Cell apoptosis was detected by flow cytometry using the Annexin V-Alexa Fluor 647/ propidium iodide (PI) apoptosis detection kit (Fcmacs Biotech, China). Briefly, cells were incubated in complete culture medium for 12 h and then exposed to TMZ (200 µM) treatment for another 48 h. Subsequently, the cells were harvested and stained according to the instructions and then analyzed using flow cytometry (FACS Canto II, Becton Dickinson). The apoptotic cells were classified as both early apoptotic (Annexin V single positive; Annexin V^+^/PI^−^) and late apoptotic/necrotic (Annexin V/PI double positive; Annexin V^+^/PI^+^).

### Cell growth inhibition assay

We conducted cytotoxicity studies in 96-well plates and determined the optimal seeding densities for each cell line to ensure exponential growth during the assay. The cells were cultured with medium containing a gradient concentration of drugs for 48 h. Cell viability was assessed using CCK8, and the optical density (OD) at 450 nm was measured with a microplate reader (Thermo Fisher Scientific). To avoid the impact of Dox spontaneous fluorescence on the results, we used a dual-wavelength detection approach (main wavelength at 450 nm and reference wavelength at 540 nm) to test the IC50 of Dox. The percentage of cell survival at each concentration was calculated using the formula: (OD _treated_/OD _untreated_) ×100. The IC_50_ value represented the drug concentration that reduced cell growth by 50%.

### Cellular uptake assay

The uptake of Dox into cells was assessed using established protocols [7]. In brief, cells were plated in 6-well plates and allowed to incubate for 24 h. Following this, the cells were treated with or without Dox (5 µM) for 3 h. Subsequently, the cells were collected and washed with cold PBS. The uptake of Dox was measured using a flow cytometer and analyzed with FlowJo VX software (FlowJo, LCC, OR, USA) based on its red fluorescence. Cells that were not exposed to Dox served as background controls. The relative mean fluorescence intensity (MFI) was calculated by comparing the MFI of Dox-positive cells to that of the blank control.

### Reverse-transcription quantitative PCR (RT-qPCR)

Total RNA was extracted using Trizol reagent (Thermo Fisher Scientific) following the manufacturer’s instructions. cDNA was synthesized using the M-MLV Reverse Transcriptase Kit (Vazyme). RT-qPCR analyses were conducted to quantify mRNA expression levels using Real SYBR Mixture (Vazyme) on a Lightcycler 480 II instrument (Roche Applied Science, USA). GAPDH served as an internal control. The specific oligonucleotide primer sequences are listed in Supplementary Table 4.

### Western blot analysis

To conduct Western blot analysis, cells were collected and lysed in RIPA lysis buffer (Cell Signaling Technology, Danvers, MA, USA). The protein concentration was measured using the BCA Protein Assay Kit (CWBIO, China). The Western blot assays were performed following a standard protocol [15]. In brief, equal amounts of proteins were loaded onto a 10% polyacrylamide gel and separated by SDS-PAGE. The samples were then transferred to PVDF membranes. After blocking with 5% non-fat milk, the membranes were incubated with primary and secondary antibodies. The target bands were visualized using chemiluminescence (Millipore, Billerica, MA, USA). The antibodies used are specified in Supplementary Table 5.

### Luciferase assay

To assess p53 transcriptional activity, we performed the luciferase assay using the Dual-Luciferase Reporter Assay Kit (Promega, Madison, WI) as per the manufacturer’s instructions. In short, TMZ-S and TMZ-R cells were plated in 24-well plates and then transfected with p53 RE-pGL4.38 to assess p53 transcriptional activity. After 24 h of transfection, the cells were lysed, and the firefly luciferase activity was measured with Renilla luciferase serving as a DNA transfection control.

### ChIP and ChIP RT-qPCR assays

The ChIP assay was performed using the Hyperactive pG-MNase CUT&RUN Assay Kit followed by RT-qPCR (Vazyme) according to the manufacturer’s instructions. Briefly, 2 × 10^7^ cells and anti-p53 antibody were used for each ChIP. Mouse IgG was applied as a negative control and spike-in DNA was used for homogenization correction. Through a protein G-fused MNase nuclease, the target protein was precisely targeted with antibody guidance, and the DNA was subsequently fragmented near the target site. After reversing cross-links and purifying the DNA, the samples were used for quantitative RT-qPCR with specific primers targeting the promoters. The primers used in this study are listed in Supplementary Table 6.

### Tumour xenografts in nude mice

Four-week-old male nude mice were obtained from Changzhou Cavens Experimental Animal Co. Ltd (Jiangsu, China) and kept in specific pathogen-free conditions. The mice were randomly assigned. For the intracranial xenograft assay, cells stably expressing the firefly luciferase gene and the indicated E2F7 OE or KD constructs were transplanted into the brain (5 × 10^5^ cells in 5 µl DMEM per mouse). After 14 days of transplantation, tumor growth was monitored via bioluminescent imaging using the IVIS Spectrum system and quantified by Living Image Software, as described previously [15]. Subsequently, the tumor-bearing mice received TMZ treatment (20 mg/kg) every 2 days for a total of 7 doses and were monitored using bioluminescent imaging until 28 days post-transplantation. At the end of the experiment, the mice were euthanized in accordance with ethical animal use guidelines. The fluorescence of each mouse before and after TMZ treatment was compared. All animal care and handling procedures adhered to the National Institutes of Health’s Guide for the Care and Use of Laboratory Animals and were approved by the Institutional Review Board of Nanjing Medical University (No. DWLS2023-24). All animal experiments were conducted by two technicians who were blinded to the treatment condition of the mice.

### Statistical analysis

All experiments were independently repeated at least three times, and the data are expressed as mean ± standard deviation (SD). The difference between 2 independent samples or among multiple groups was determined by the student *t*-test or one-way ANOVA followed by a Student-Newman-Keuls multiple comparison test (SNK test) respectively. Survival analyses were performed using the Kaplan-Meier method with the log-rank test. The correlation of gene expression was evaluated using Spearman’s rank correlation analysis. Statistical significance was set at *p* < 0.05. The Statistical Package for Social Science (SPSS) 20.0 package (IBM, USA) and GraphPad Prism 8.0 software (GraphPad, USA) were used for all statistical analyses and data graphing respectively.

## Results

### Elevated E2F7 is correlated to chemoresistance and predicts adverse prognosis of GBM

**Fig. 1:**
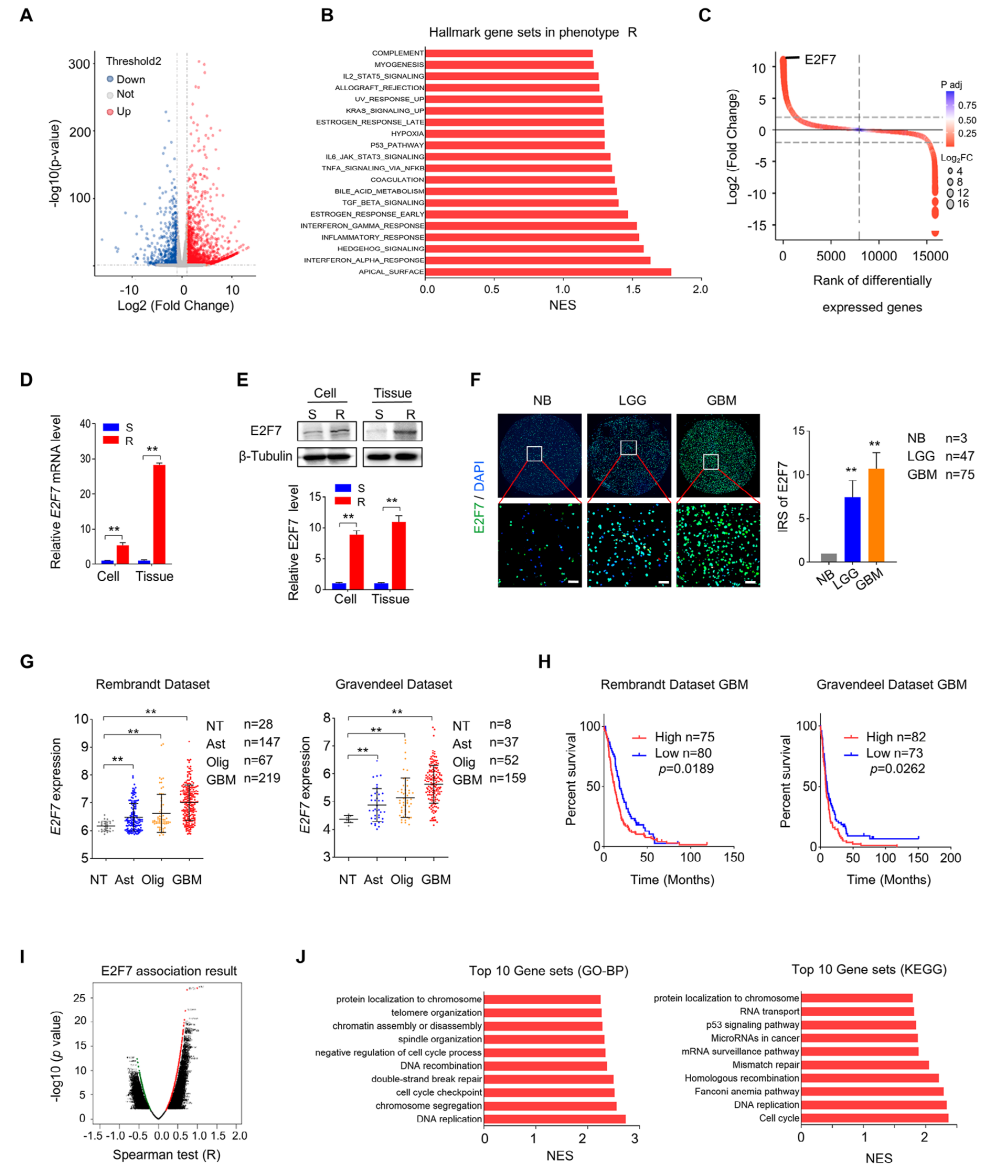
Elevated expression of E2F7 correlates with TMZ resistance and predicts an adverse prognosis. **(A)** Volcano plot analysis illustrating the differentially expressed genes in TMZ-sensitive (TMZ-S) and TMZ-resistance (TMZ-R). **(B)** The top enriched gene categories were identified by GenePattern based on genes positively correlated with TMZ-R. A NES value of 1.0 and *p* < 0.05 were used as visual thresholds. **(C)** Rank diagram displaying the top 50 differentially expressed genes in TMZ-R and TMZ-S tumors at the mRNA level. **(D, E)** The expression of E2F7 in TMZ-S and TMZ-R tissue measured by RT-qPCR (C) and Western blot (D) (*n* = 3, **p < 0.01). β-Tubulin served as a loading control. **(F)** Representative IF images of the E2F7 expression in the same glioma tissue sections (scale bars, 50 μm). The immunoreactivity score (IRS) of E2F7 is displayed on the right side. NB, Normal Brain; LGG, Lower Grade Glioma; GBM, Glioblastoma multiforme. **(G)** The expression of E2F7 in different grades of gliomas in the Gliovis-Rembrandt and Gliovis-Gravendeel databases. NT, non-tumor brain tissues; Ast, astrocyte; Olig, oligodendrocyte; GBM, glioblastoma multiforme. **(H)** Overall survival analysis based on E2F7 expression in the indicated GBM datasets. **(I)** GSEA analysis according to TCGA_GBM data derived from Linked-Omic. **(J)** Top 10 gene sets analyzed by GO and KEGG.

In the previous study, we developed an acquired TMZ resistant in vivo model, which enabled passable and stable resistant tumors (TMZ-R) and primary cells [7]. Whole transcriptome sequencing was performed on the derived TMZ-R and corresponding sensitive (TMZ-S) xenografts. The volcano plot showed the distribution of differentially regulated genes between TMZ-R and TMZ-S tumors (Fig. 1A). According to GSEA, the upregulated genes in TMZ-R tumors were enriched in various Hallmark gene sets, including apical surface, interferon response, p53 pathway, and cytokine associated pathways (Fig. 1B). Among these, the p53 pathway attracted our attention. The top 50 differentially regulated genes between TMZ-R and TMZ-S tumors involving in p53 pathway were collected, and E2F7, an atypical member of the E2F transcription factor, ranked first among upregulated genes in TMZ-R tumors (Fig. 1C). The increase of E2F7 in TMZ-R tumors and derived primary cells was further confirmed by RT-qPCR (Fig. 1D) and western blot (Fig. 1E). To explore the clinical relevance of E2F7 in glioma, we further analyzed its expression in local clinical samples and public datasets. Immunofluorescence (IF) staining of glioma tissue array derived from an in-house cohort revealed that E2F7 protein is significantly upregulated in tumors, and most significantly in GBMs (Fig. 1F). According to the results derived from the public glioma datasets, E2F7 was confirmed as a highly expressed gene in gliomas, and was correlated with glioma grade (Fig. 1G). Overall survival analysis indicated that higher levels of E2F7 predicted worse survival outcome for GBM patients (Fig. 1H), suggesting E2F7 is potentially involved in tumorigenesis and recurrence of GBM. Subsequently, genes whose expression correlated with E2F7 in TCGA_GBM data from Linked-Omic were collected (Fig. 1I) and GSEA analysis was performed. It showed that the function of genes positively correlated with E2F7 were closely related to the regulation of DNA replication, cell cycle, DNA damage and repair (Fig. 1J). Together, these results suggest that E2F7 is a carcinogenic gene and potentially contributes to the chemoresistance of GBM.

### E2F7 upregulation is required for TMZ resistance of GBM cells

**Fig. 2:**
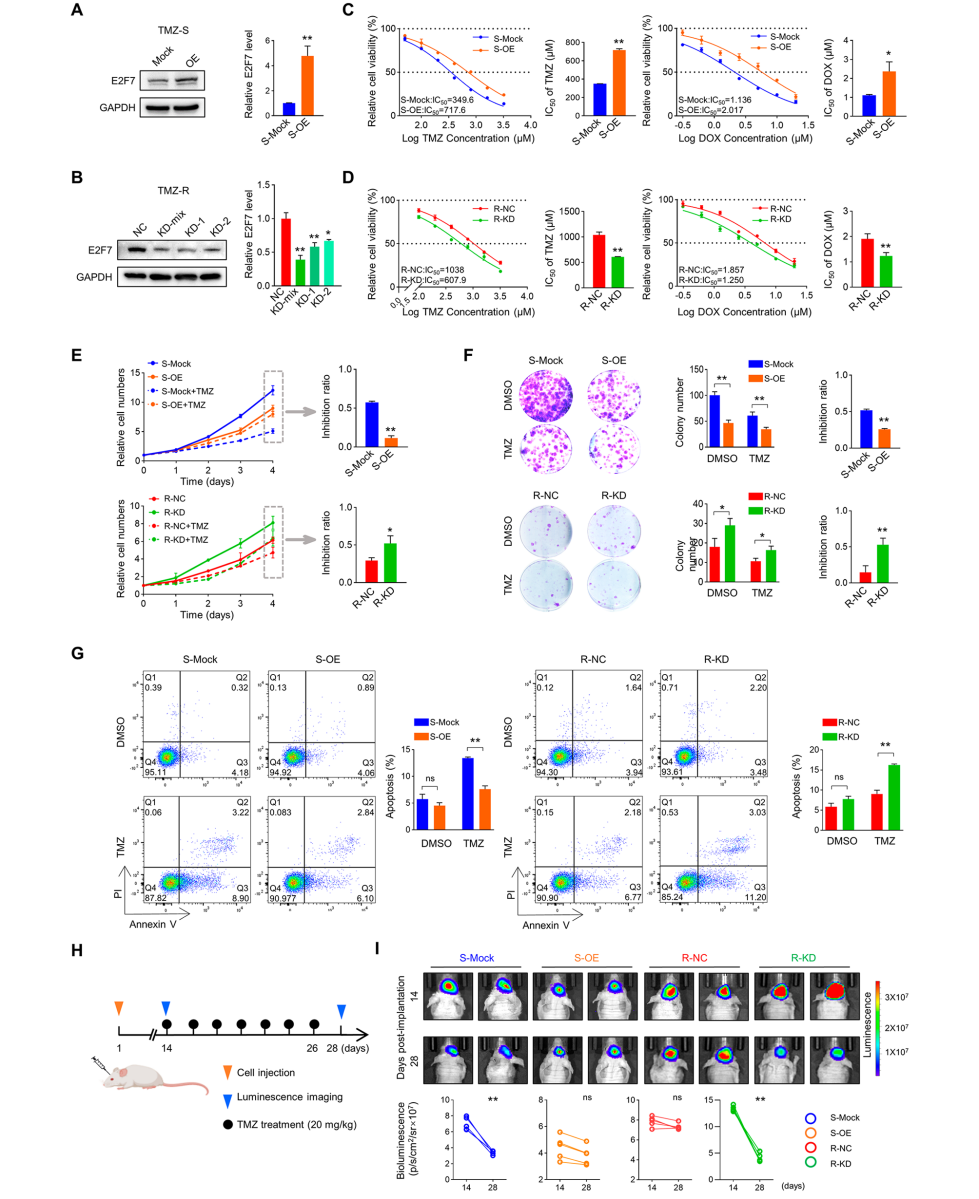
E2F7 upregulation is required for TMZ resistance in GBM cells. **(A-B)** Confirmation of E2F7 overexpression in TMZ-S cells and siRNA knockdown in TMZ-R cells by Western blot. GAPDH served as a loading control (*n* = 3, *p < 0.05, **p < 0.01). **(C-D)** IC_50_ assay indicating different drug responses in cells with varying levels of E2F7 expression after 48 h of treatment with TMZ or Dox (*n* = 3, *p < 0.05, **p < 0.01). **(E)** CCK-8 assay indicating the cell viability of indicated cells with or without TMZ treatment (200 µM). The growth inhibition ratio of TMZ is presented on the right panel (*n* = 6, *p < 0.05, **p < 0.01). **(F)** Colony formation of indicated cells with or without TMZ treatment (200 µM). The colony number and colony inhibition of TMZ are presented on the right panel (*n* = 3, *p < 0.05, **p < 0.01). **(G)** Apoptosis analysis by flow cytometry of indicated cells with or without TMZ treatment (200 µM, 48 h). The apoptosis rate in the indicated groups is presented on the right panel (*n* = 3, **p < 0.01). **(H)** Schematic diagram of in vivo experimental process. **(I)** Representative bioluminescence images (BLI) of the indicated groups taken at 14- and 28-days post-transplantation. The relative BLI quantification is presented on the lower panel (*n* = 5, **p < 0.01)

Next, we explored whether upregulation of E2F7 contributed to acquired resistance to TMZ in GBM. For this purpose, cells derived from TMZ-S xenografts were overexpressed (OE) with ectopic E2F7 (Fig. 2A), while cells from TMZ-R xenografts were treated with siRNA constructs to achieve E2F7 knockdown (KD) cells (Fig. 2B). Due to their similar KD efficiency, the two siRNAs were mixed in equal proportions (KD-mix) for subsequent experiments. First, an IC_50_ assay of TMZ and Dox was performed to examine whether E2F7 affects multidrug resistance, an outstanding characteristic of TMZ-R xenografts and cells [7]. As shown in Fig. 2C, TMZ-S cells acquired a certain chemotherapy resistance upon ectopic E2F7 introduction. However, E2F7 KD significantly impaired the response to chemotherapy in TMZ-R cells, as confirmed by a decreased IC_50_ (Fig. 2D). To further document the effects of E2F7 on chemosensitivity of GBM cells, a relatively lower concentration of TMZ (200 µM) was used to determine the cellular response to mild chemotherapy. The cell growth assay indicated that this concentration sustained growth inhibition of TMZ-S cells, and had a weaker effect on TMZ-R cell growth (Fig. 2E, left panel). By comparing the fold change of TMZ treatment to vehicle treatment (growth inhibition) between the indicated groups at the final time point, a significant growth inhibition in TMZ-S cells was observed with E2F7 OE or TMZ-R cells with E2F7 KD upon TMZ treatment (Fig. 2E, right panel). The observations in colony formation assay corroborated that E2F7 functions in response to chemotherapy (Fig. 2F). An unexpected observation in Fig. 2E and F was that E2F7 OE impaired the cell proliferation of TMZ-S cells, while E2F7 KD induced an increased cell growth capacity of TMZ-R cells. The inconsistent growth abilities of TMZ-S and TMZ-R cells in vivo and in vitro make it difficult to draw a clear conclusion about the function of E2F7 in GBM tumorigenicity. Therefore, we continued to focus on the role of E2F7 in the development of chemotherapy resistance. According to the apoptosis assay, E2F7 OE or KD fail to affect the cell apoptosis of TMZ-S or TMZ-R cells, respectively, in the absence of TMZ. However, upon TMZ treatment, E2F7 OE provides protection in TMZ-S cells, while E2F7 KD restores sensitivity in TMZ-R cells (Fig. 2G). To further document the function of E2F7 in TMZ resistance of GBM, an intracranial xenograft model was performed in nude mice using luciferase labeled TMZ-S and TMZ-R cell. The tumors were allowed to grow freely for 14 days after cell-implantation, and then received a course of intraperitoneal TMZ or normal saline at a lower dose (20 mg/kg) once every 2 days for 7 consecutive injections (Fig. 2H). Tumor size was monitored by luminescence imaging before and after TMZ treatment. As shown in Fig. 2I, the tumor size of TMZ-S tumors significantly decreased after TMZ treatment, while TMZ therapy failed to exhibit its inhibition in tumors expressing ectopic E2F7. According to the observation in TMZ-R tumors, E2F7 KD significantly promoted the chemosensitivity. Collectively, these findings revealed that E2F7 is required for sustained chemoresistance of GBM cells.

### E2F7 upregulation impairs drug uptake and DNA damage induced by chemotherapy

**Fig. 3:**
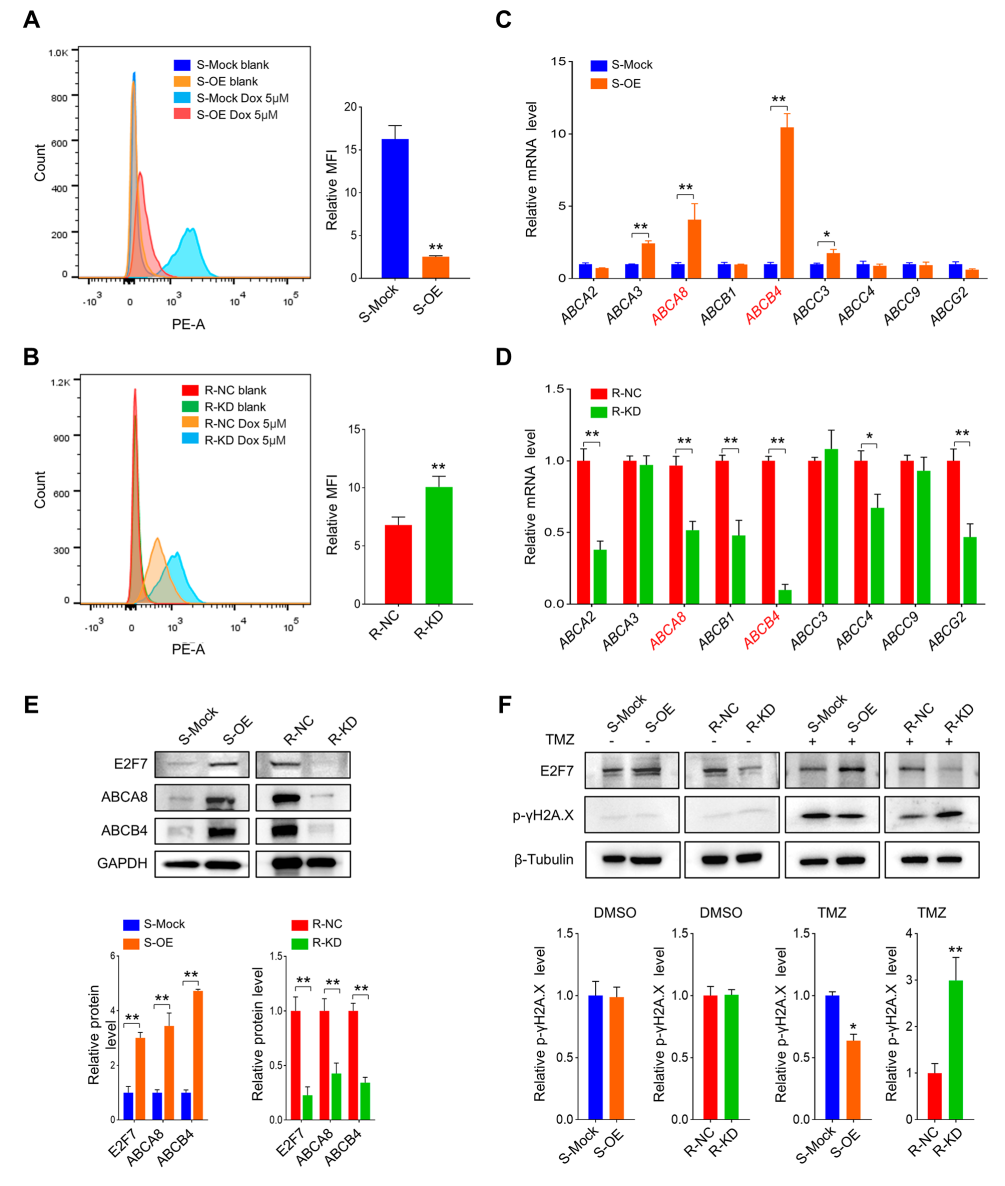
E2F7 contributes to drug efflux and decreases DNA damage. **(A-B)** Flow cytometry assay (left panel) and statistical analysis of mean fluorescence intensity (MFI) (right panel) showing the uptake of Dox in cells with varying levels of E2F7 expression (*n* = 3, **p < 0.01). **(C-D)** RT-qPCR assay measuring the expression of *ABCA2*, *ABCA3*, *ABCA8*, *ABCB1*, *ABCB4*, *ABCC3*, *ABCC4*, *ABCC9* and *ABCG2* mRNA in the indicated cells (*n* = 3, *p < 0.05, **p < 0.01). **(E)** Western blot assay showing the expression of ABCA8 and ABCB4 protein in the indicated cells, GAPDH served as a loading control. **(F)** Western blot assay showing the expression of γH2A.X in the indicated groups with or without treatment of TMZ (200 µM for 48 h), β-Tubulin served as a loading control

Due to the acquired TMZ resistance of TMZ-R cells with impaired drug uptake [16], we inquired whether E2F7 upregulation contributes this functional characteristic. According to established method [16], we used Dox, a small molecule drug with red fluorescence, to investigate the effect of E2F7 on drug accumulation in cells. Flow cytometry assay showed that the uptake of Dox was significantly decreased in E2F7 OE cells and increased in E2F7 KD cells compared to their respective controls (Fig. 3A and B). The multi-drug resistance of TMZ-R is associated with upregulation of ATP binding cassette ABC transporters [7], so we subsequently investigated the effect of E2F7 on these transporters. The qRT-PCR assay indicated that two typical ABC transporters, *ABCA8* and *ABCB4* [17, 18] were positively regulated by manipulation of E2F7 expression (Fig. 3C and D). The regulation of E2F7 on the expression of ABCA8 and ABCB4 was further confirmed by Western blot analysis (Fig. 3E). By detecting γH2A.X, an indicator of DNA double-strand breaks, we found that overexpression of E2F7 reduced DNA damage in TMZ-S cells induced by TMZ, while knockdown of E2F7 restored TMZ-genotoxicity in TMZ-R cells (Fig. 3F). In summary, E2F7 hinders drug uptake and subsequent genotoxicity by increasing the expression of ABC transporters.

### E2F7 is a p53 target gene and is responsible for TMZ-mediated resistance

**Fig. 4:**
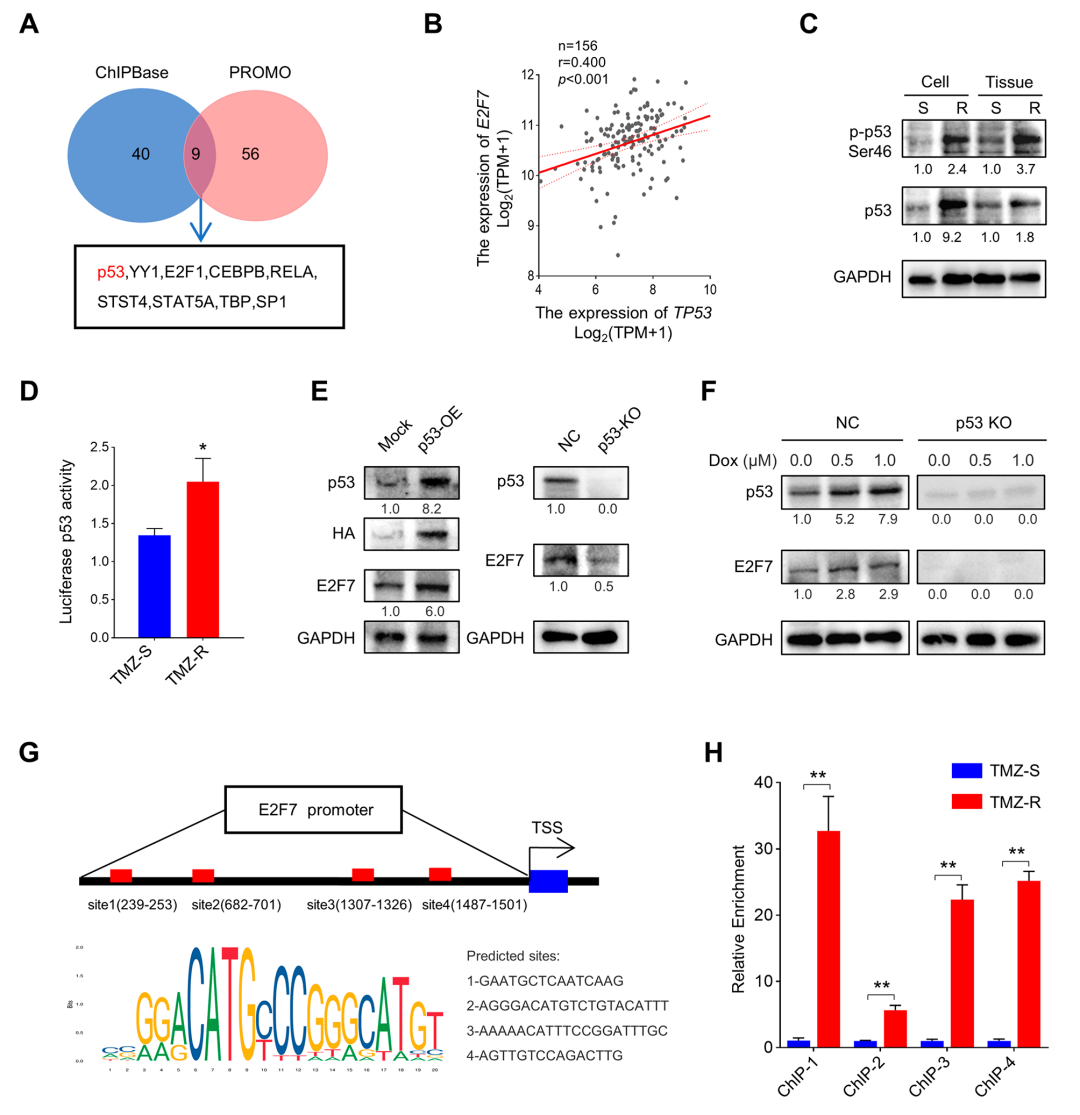
E2F7 stabilizes resistance in GBM cell lines in a p53-dependent manner. **(A)** Schematic representation of the candidate transcription factor screening approach, using ChIPBase and PROMO databases. **(B)** Correlation between E2F7 and TP53 in TCGA_GBM database (Spearman’s rank correlation test). **(C)** The p53 and phosphorylation p53 (Ser46) expression in TMZ-S and TMZ-R tissue and tissue derived cells, GAPDH served as a loading control. The quantification of p-p53 and p53. **(D)** Relative luciferase activity of p53 in TMZ-S and TMZ-R cells (*n* = 3, *p < 0.05). **(E)** Western blot analysis of E2F7 and p53 expression in the indicated TMZ-S cells. **(F)** Western blot analysis of E2F7 and p53 expression in the indicated TMZ-S cells treated with Dox. **(G)** Predicted P53-binding sites in the promoter region of E2F7 using JASPAR database. TSS: transcriptional start site. **(H)** The relative enrichment compared to that of input of TMZ-S and TMZ-R cells at the potential p53 binding sites (*n* = 3, **p < 0.01)

To further explore the potential mechanism underlying the increased expression of E2F7 in TMZ-R cells, we explored the PROMO and ChIPBase databases. The interactome analysis predicted nine transcription factors that may regulate E2F7, among which, p53 aroused our interest (Fig. 4A). As mentioned above, the p53 pathway was enriched in TMZ-R cells, and analysis of the TCGA_GBM database showed a positive correlation between *TP53* and *E2F7* (Fig. 4B). Therefore, we considered that p53 may mediate the high expression of E2F7 in TMZ-R cells. Western blot analysis revealed that p53 was highly expressed in TMZ-R tissue and cells. More importantly, p53 activity was also increased in TMZ-R cells, as confirmed by the expression of p-p53 (ser46) and a luciferase experiment (Fig. 4C and D). To further investigate the regulatory relationship between p53 and E2F7, lentiviruses for p53 overexpression (OE) and CRISPR/Cas9-meditated p53 knockout (KO) were constructed. TMZ-S cells stably expressing different levels of p53 were obtained. Western blot analysis showed that the expression of E2F7 changed synchronously with p53 (Fig. 4E). Interestingly, E2F7 increased accompanied by increased p53 in a dose-dependent manner, when treated with Dox in TMZ-S cells, while this trend was not observed in p53 KO TMZ-S cells (Fig. 4F). Using the JASPAR database, p53 binding sites on the E2F7 promoter were predicted (Fig. 4G), and verified by ChIP-qPCR (Fig. 4H). Collectively, these data suggested that the up-regulation of E2F7 is dependent on the activity of p53.

## Discussion

GBM is an aggressive and incurable tumor with a bleak clinical prognosis. The primary treatments in the clinic include surgery, perioperative chemotherapy, and chemoradiotherapy. TMZ is commonly used as a chemotherapeutic agent and has shown significant improvement in patient survival rates and time [19, 20]. However, at least 50% of patients treated with TMZ do not respond to the treatment, suggesting that chemotherapy resistance remains a great challenge in clinical treatment [4]. Therefore, it is crucial to gain a systematic understanding of the molecular mechanisms that contribute to TMZ resistance in order to improve the poor prognosis of GBM patients.

The E2F family is a class of transcription factors that are involved in various cellular processes, including cell cycle regulation, DNA repair, and cellular quiescence [9, 21]. It has been reported that they play an important role in cancer progression, for example, E2F1 is considered to be an important driver of tumor growth, including gastric cancer, breast cancer, and melanoma [22, 23], in cancer stem cells (CSCs), E2Fs are key players in tumor development, metastasis, drug resistance and recurrence [24]. Unlike E2F1, E2F7, an atypical E2F factor, plays different roles in individual cancer types. For example, E2F7 acts as a competitive factor of E2F1, inhibiting transcriptional activation and tumor promotion induced by E2F1 [25]. On the other hand, E2F7 is significantly increased during the formation of tumor spheres in liver cancer stem cells (LCSCS) and plays a role in promoting resistance of hepatocellular carcinoma to rapamycin [26], estrogen receptor (ER)-positive breast cancer against tamoxifen (TAM) [27], and neck squamous cell carcinoma (HNSCC) cells against doxorubicin [28].

This study demonstrated that there is a high expression of E2F7 in GBM, and its elevated expression is associated with poor prognosis. Furthermore, we have discovered that E2F7 is expressed abnormally in the cells and tissues of our established TMZ-resistant GBM model. Through our in vitro and in vivo experiments, we have confirmed that overexpression of E2F7 promotes chemoresistance in TMZ-S cell, and E2F7 knockdown partially restores sensitivity in TMZ-R cells. Unexpectedly, in the absence of TMZ, overexpressing E2F7 inhibited the proliferation of TMZ-S cells, while knocking down E2F7 promoted the proliferation of TMZ-R cells with no impact on apoptosis. These results seemed to contradict the conclusion that E2F7 is associated with poor prognosis in GBM patients. However, an increasing number of studies indicate that many genes play dichotomous and contradictory roles depending on their environment. For example, the Kelch-like ECH-associated protein 1 (KEAP1)/nuclear factor erythroid 2-related factor 2 (NRF2) pathway serves as a physiological defense mechanism against xenobiotics and reactive oxygen species. Nevertheless, the activation of NRF2 confers significant benefits to tumors, as it reconfigures metabolic processes to facilitate proliferation, mitigates diverse stressors, and facilitates evasion of the immune system [29]. In our previous research, we discovered that TCF4N (β-Catenin interacting transcription factor TCF7L2) plays a role in promoting GBM tumorigenesis, stem cell differentiation, and chemosensitivity [30]. Even the well-known tumor inhibitor p53, in some cases, promotes tumor growth [31, 32] and chemosensitivity [33, 34]. Previous studies have shown that E2F7 is a key component of a negative feedback loop required to turn off transcription of E2F-driven G1/S target genes, thus allowing progression through the cell cycle [35]. In this study, TMZ causes DNA damage, leading to cellular stress, which enables tumor cells to survive in stressful conditions and mediate drug resistance.

Drug resistance mechanisms in tumor cells often involve dysregulation of the cell cycle, enhanced drug efflux, and weakened drug absorption. Our study also revealed that overexpression of E2F7 leads to drug aggregation in TMZ-S cells. Subsequent studies have indicated that E2F7 is responsible for regulating the expression of ABCA8 and ABCB4, members of the ATP-binding cassette (ABC) transporters, which are widely recognized for their involvement in drug resistance [36–38]. Although we have extensively searched through public databases GTRD (biouml.org) and UCSC, as well as conducted a comprehensive review of the literature, we have not been able to find any evidence to support the direct control of *ABCA8* and *ABCB4* transcription by E2F7. Therefore, we have proposed the hypothesis that E2F7 may regulate ABCA8 and ABCB4 indirectly through other pathways, which will be the primary focus of our future research. These observations, as well as E2F7 overexpression impairing chemotherapy induced DNA damage accumulation in cells, indicate that E2F7 is a key manipulator in promoting chemotherapy resistance.

E2F7 is involved in the negative regulation of genes controlling DNA repair pathways, controlling cellular recovery during an ongoing DNA damage response. One study showed that E2F7 plays a p53-independent role in attenuating of DNA repair function through transcriptional repression of target genes required for the timely regulation of replication fork-associated DNA damage repair [39]. However, several reports have shown that the upregulation of E2F7 in response to DNA damage is dependent on p53, with E2F7 being a direct target for transcriptional activation by p53 [12, 40]. E2F7 expression and cell-cycle target gene repression have been previously linked to p53 after DNA damage caused by topoisomerase inhibitors [41]. In this study, we found that p53 expression and p53 phosphorylation were significantly increased in TMZ-R cells, and the changes of E2F7 under drug stimulated conditions were regulated by p53 in p53 WT cells. This was validated by luciferase reporter assays and ChIP analysis, which showed that p53 mechanically promoted E2F7 transcription. Generally, p53 is frequently mutated in GBM, and the impact of mutant p53 on TMZ resistance is complex. For example, the suppression of MGMT reporter gene activity predicts a higher efficacy of TMZ in p53 WT cells [8, 42], while circ_0072309 has been identified as a regulator of TMZ sensitivity in p53 WT cells, but not in p53 mutant GBM cells [43]. Overall, these findings provide additional insights into the regulatory mechanisms underlying E2F7 expression in response to TMZ resistance.

## Conclusions

In summary, the present study discloses that continuous treatment with TMZ leads to the phosphorylation and activation of p53, which in turn upregulates the transcription of E2F7 and confers chemoresistance to GBM cells by inhibiting drug uptake and DNA damage. These findings highlight the significant connection between sustained p53 activation and GBM chemoresistance, offering potential for new strategies to overcome this resistance.

### Electronic supplementary material

Below is the link to the electronic supplementary material.

Supplementary Material 1

Supplementary Material 2

## Data Availability

The datasets used and analysed during the current study are available on reasonable request from the corresponding author. Direct web links of public datasets about as follows:GSEA/MSigDB:https://www.gsea-msigdb.org/gsea/msigdb/index.jsp;GlioVis: http://gliovis.bioinfo.cnio.es/;Metascape:https://metascape.org/;LinkedOmics:https://www.linkedomics.org/;ChIPBase: https://rnasysu.com/chipbase3/index.php; PROMO: https://alggen.lsi.upc.es/;TCGA: https://www.cancer.gov/;JASPASdatabases:https://jaspar.genereg.net/.

## Abbreviations

GBM: Glioblastoma multiforme
TMZ: Temozolomide
E2F7: E2F Transcription factor 7
TMZ-R: TMZ-resistance
TMZ-S: TMZ-sensitive
MGMT: O^6^-methylguanine-DNA methyltransferase
IHC: immunohistochemistry
HRP: secondary-horseradish peroxidase
DAPI: 4’,6-diamidino-2-phenylindole
DMEM: Dulbecco’s Modified Eagle’s Medium
CRISPR/Cas9: Clustered Regularly Interspaced Short Palindromic Repeats
EdU: 5-ethynyl-2-deoxyuridine
FACS Canto II: Fluorescence-activated cell sorting
FITC: Annexin V-Fluorescein isothiocyanate
PI: Propidium iodide
SPSS: Statistics Package for Social Science
GSEA: Gene set enrichment analysis
GO: Gene ontology
KEGG: Kyoto encyclopedia of genes and genomes
LGG: Lower grade gliomas
OE: Over expression
KD: Knock down
KO: Knock out
WT: Wild type
Dox: doxorubicin
ABCA8: ATP binding cassette A8
ABCB4: ATP binding cassette B4
MDR: Multidrug resistance
