# Supplementary material for: p53/E2F7 axis promotes temozolomide chemoresistance in glioblastoma multiforme
Source: BMC Cancer. 2024 Mar 7;24:317. doi: 10.1186/s12885-024-12017-y (PMC10921682; doi:10.1186/s12885-024-12017-y)

Figure 1E.

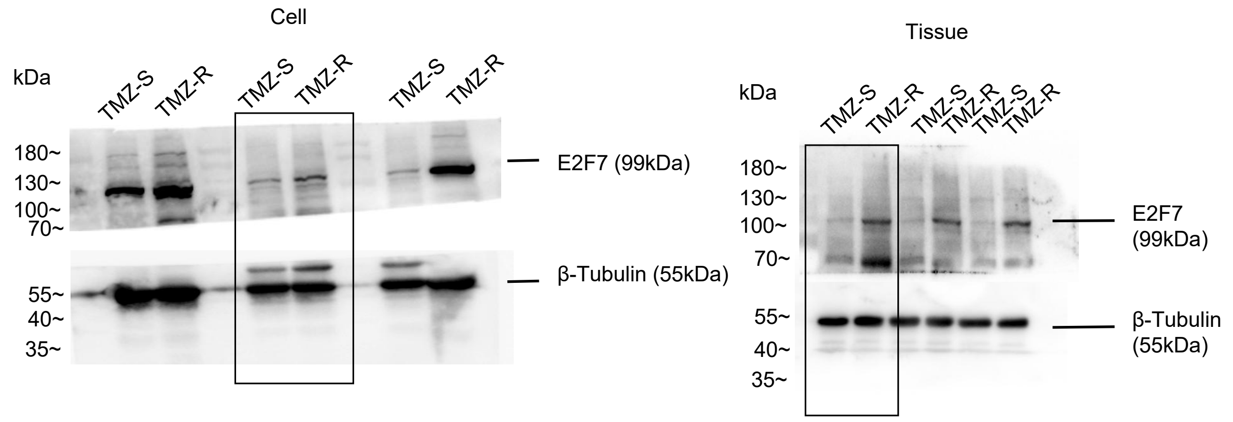

Figure 2A.

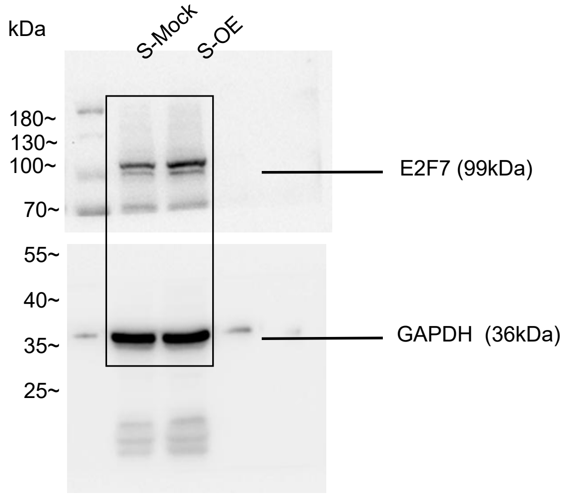

Figure 2B.

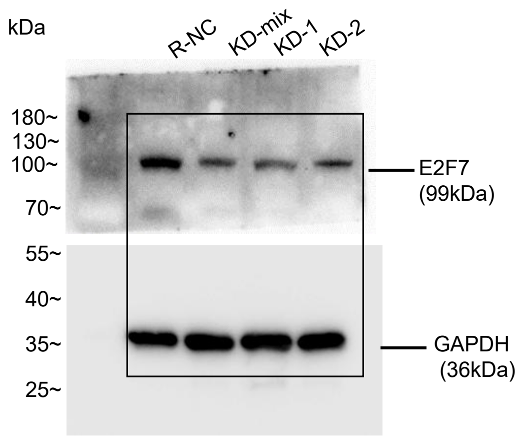

Figure 3E left

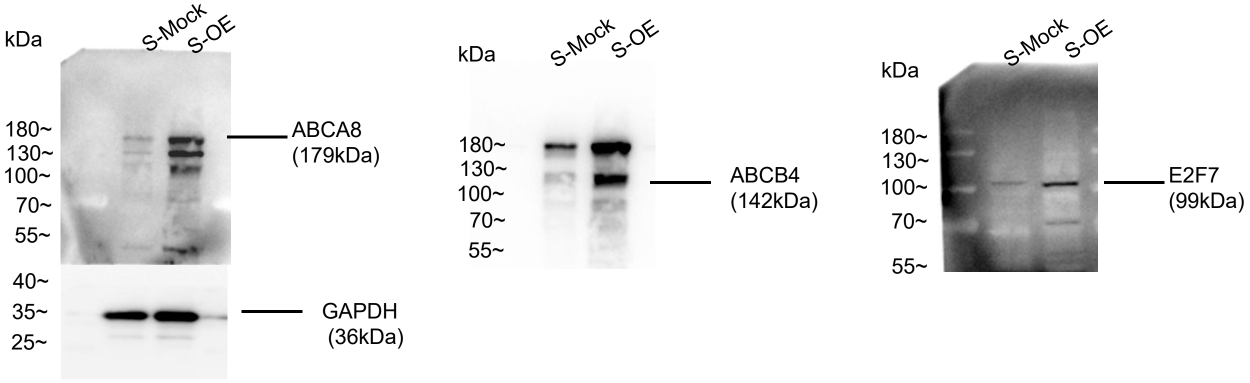

Figure 3E right

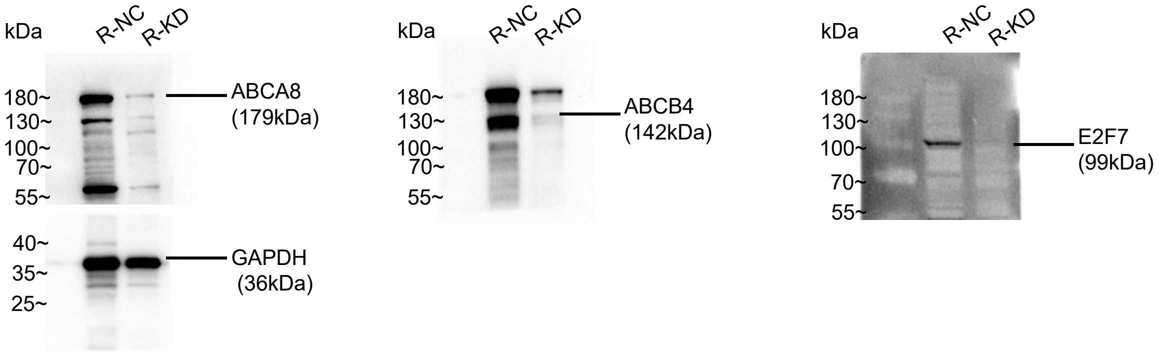

Figure 3F left

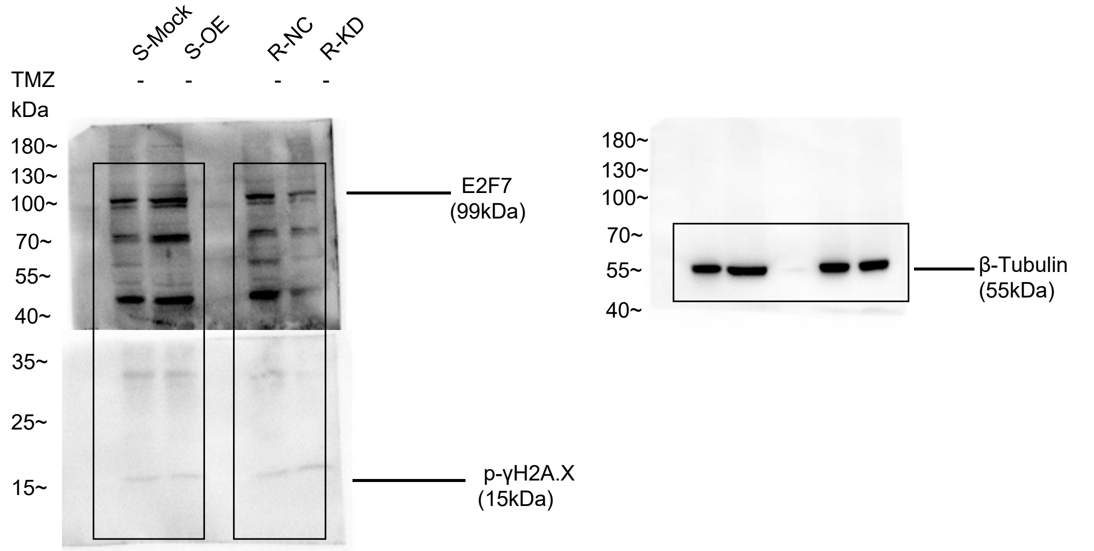

Figure 3F right

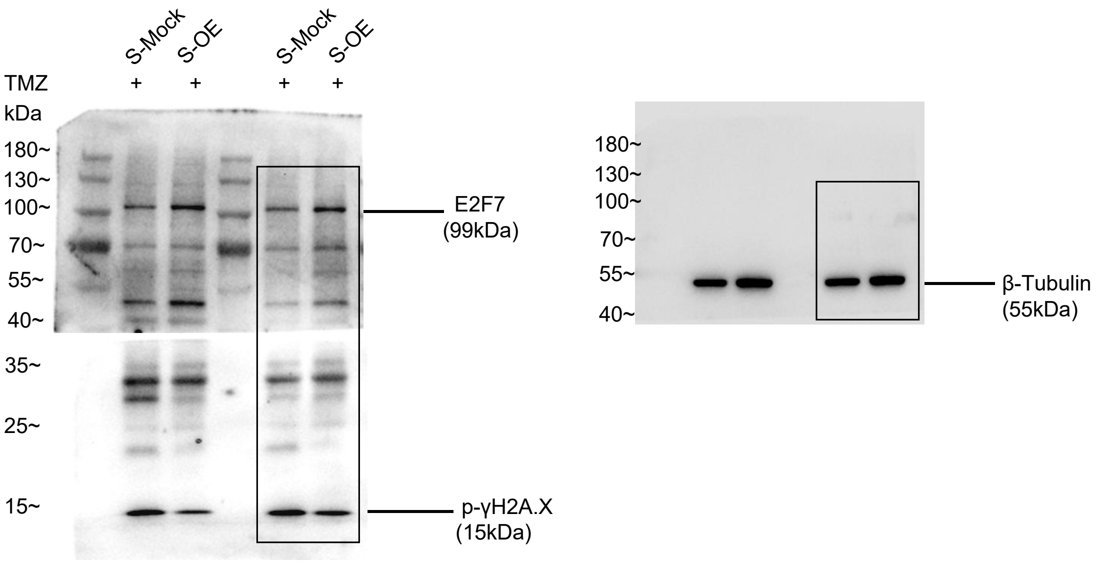

Figure 3F right

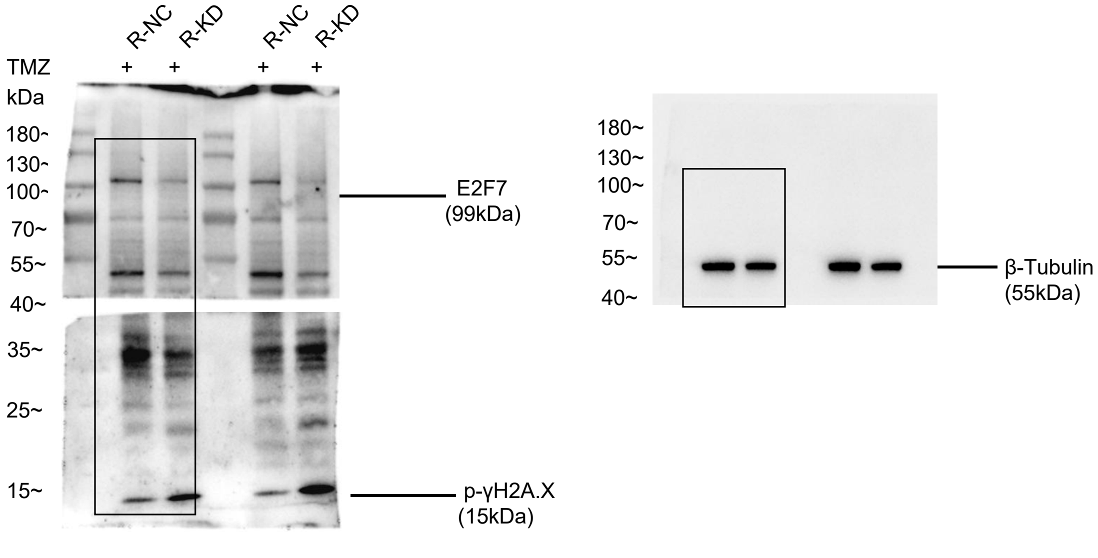

Figure 4C

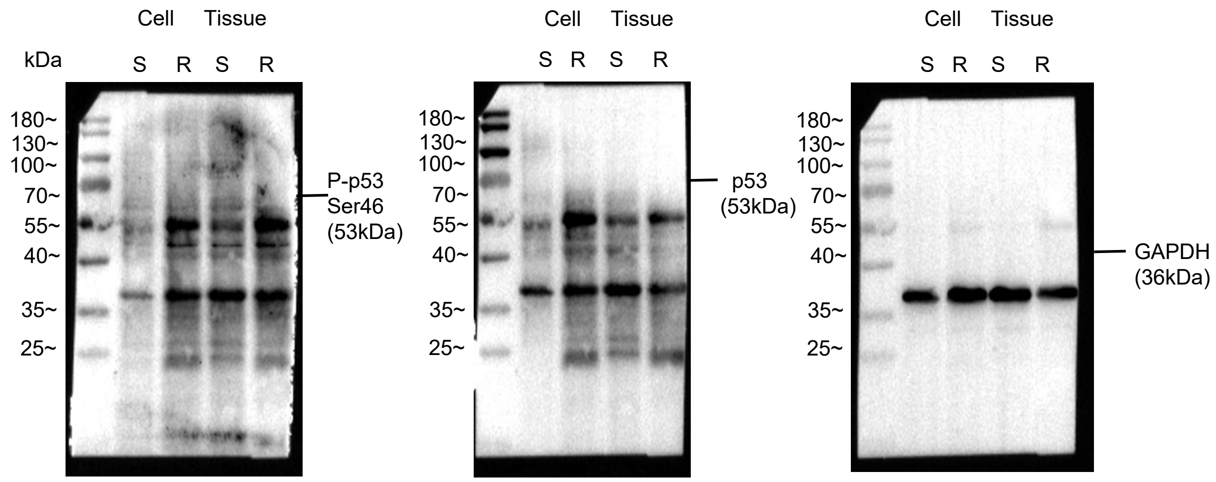

Figure 4E left

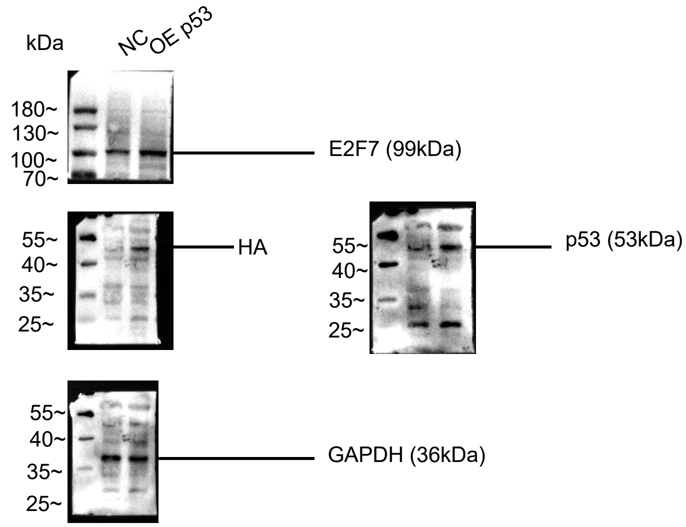

Figure 4E right

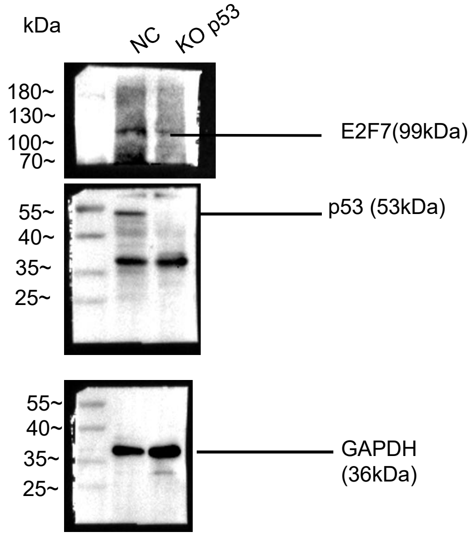

Figure 4F left

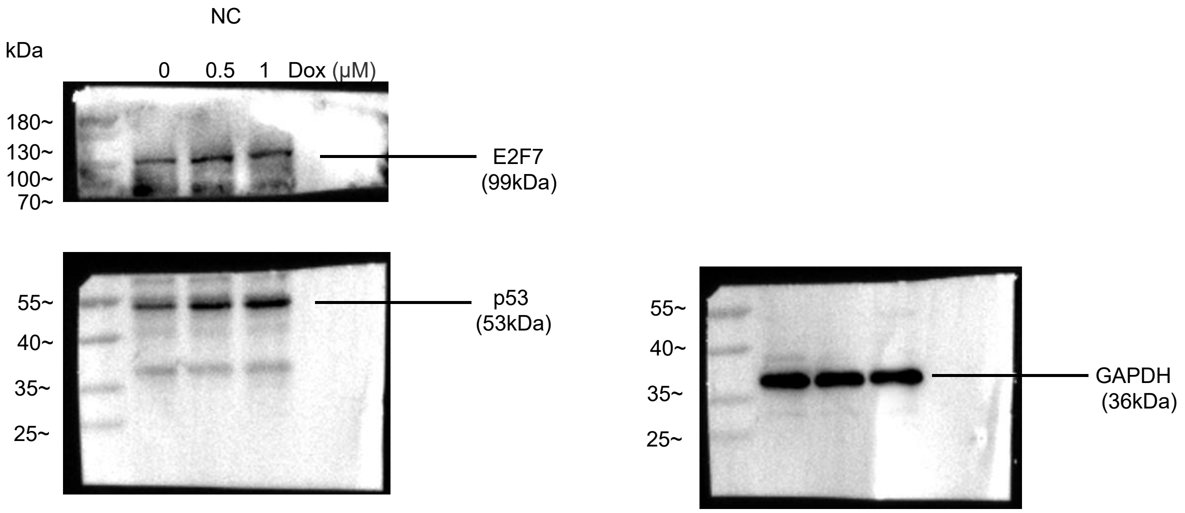

Figure 4F right

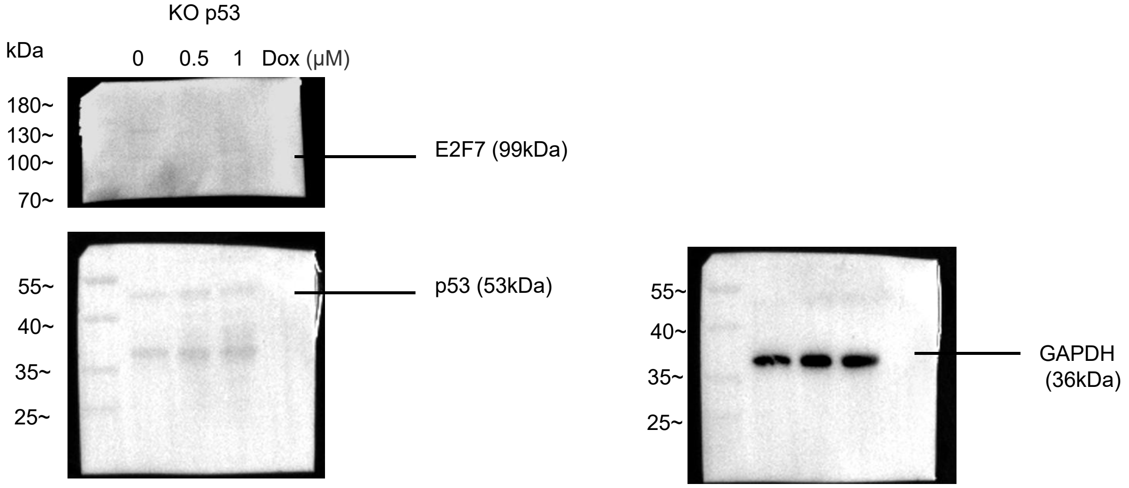

Supplement: Supplementary file 1 — Supplementary Material 1 [file 12885_2024_12017_MOESM1_ESM.pdf]
