# Supplementary material for: p53/E2F7 axis promotes temozolomide chemoresistance in glioblastoma multiforme
Source: BMC Cancer. 2024 Mar 7;24:317. doi: 10.1186/s12885-024-12017-y (PMC10921682; doi:10.1186/s12885-024-12017-y)
Supplement: Supplementary file 2 — Supplementary Material 2 [file 12885_2024_12017_MOESM2_ESM.doc]

**Supplementary Table 1. Clinical pathological information of human samples**

| **No.** | **Gender** | **Age** | **Pathogenic type** | **Pathology grade** | **Proportion score of E2F7** | **Mean fluoresecnce intensity score of E2F7** | **IRS of E2F7** |
| --- | --- | --- | --- | --- | --- | --- | --- |
| 1 | Male | NA | NB | NA | 1 | 1 | 1 |
| 2 | Male | 22 | NB | NA | 1 | 1 | 1 |
| 3 | Male | 21 | NB | NA | 1 | 1 | 1 |
| 4 | Male | 40 | LGG | Ⅰ | 3 | 2 | 6 |
| 5 | Female | 35 | LGG | Ⅰ | 3 | 2 | 6 |
| 6 | Female | 21 | LGG | Ⅰ | 3 | 2 | 6 |
| 7 | Male | 16 | LGG | Ⅰ | 3 | 2 | 6 |
| 8 | Female | 19 | LGG | Ⅰ | 4 | 2 | 8 |
| 9 | Male | 38 | LGG | Ⅱ | 2 | 2 | 4 |
| 10 | Female | 43 | LGG | Ⅱ | 4 | 2 | 8 |
| 11 | Male | 51 | LGG | Ⅱ | 4 | 2 | 8 |
| 12 | Male | 16 | LGG | Ⅱ | 4 | 2 | 8 |
| 13 | Male | 14 | LGG | Ⅱ | 3 | 2 | 6 |
| 14 | Female | 52 | LGG | Ⅱ | 2 | 2 | 4 |
| 15 | Male | 35 | LGG | Ⅱ | 4 | 2 | 8 |
| 16 | Female | 16 | LGG | Ⅱ | 4 | 2 | 8 |
| 17 | Male | 19 | LGG | Ⅱ | 4 | 2 | 8 |
| 18 | Male | 36 | LGG | Ⅱ | 3 | 2 | 6 |
| 19 | Male | 37 | LGG | Ⅱ | 4 | 2 | 8 |
| 20 | Male | 26 | LGG | Ⅱ | 3 | 2 | 6 |
| 21 | Male | 45 | LGG | Ⅱ | 3 | 2 | 6 |
| 22 | Female | 23 | LGG | Ⅱ | 4 | 2 | 8 |
| 23 | Female | 36 | LGG | Ⅱ | 3 | 2 | 6 |
| 24 | Male | 33 | LGG | Ⅱ | 3 | 2 | 6 |
| 25 | Female | 46 | LGG | Ⅱ | 4 | 2 | 8 |
| 26 | Male | 46 | LGG | Ⅱ | 3 | 2 | 6 |
| 27 | Female | 11 | LGG | Ⅱ | 3 | 2 | 6 |
| 28 | Male | 43 | LGG | Ⅱ | 4 | 2 | 8 |
| 29 | Male | 34 | LGG | Ⅱ | 4 | 2 | 8 |
| 30 | Male | 40 | LGG | Ⅱ | 3 | 2 | 6 |
| 31 | Male | 47 | LGG | Ⅱ | 4 | 2 | 8 |
| 32 | Female | 72 | LGG | Ⅱ | 2 | 2 | 4 |
| 33 | Male | 75 | LGG | Ⅱ | 4 | 2 | 8 |
| 34 | Female | 40 | LGG | Ⅱ | 4 | 2 | 8 |
| 35 | Male | 37 | LGG | Ⅱ | 4 | 2 | 8 |
| 36 | Male | 67 | LGG | Ⅱ | 4 | 2 | 8 |
| 37 | Female | 47 | LGG | Ⅱ | 4 | 2 | 8 |
| 38 | Male | 36 | LGG | Ⅱ | 4 | 2 | 8 |
| 39 | Male | 44 | LGG | Ⅱ | 4 | 2 | 8 |
| 40 | Female | 50 | LGG | Ⅱ | 4 | 2 | 8 |
| 41 | Female | 40 | LGG | Ⅱ | 4 | 2 | 8 |
| 42 | Male | 40 | LGG | Ⅱ | 2 | 2 | 4 |
| 43 | Male | 47 | LGG | Ⅱ-Ⅲ | 4 | 3 | 12 |
| 44 | Male | 48 | LGG | Ⅱ-Ⅲ | 4 | 2 | 8 |
| 45 | Female | 35 | LGG | Ⅱ-Ⅲ | 4 | 3 | 12 |
| 46 | Female | 39 | LGG | Ⅱ-Ⅲ | 4 | 2 | 8 |
| 47 | Female | 34 | LGG | Ⅱ-Ⅲ | 4 | 2 | 8 |
| 48 | Male | 48 | LGG | Ⅱ-Ⅲ | 4 | 3 | 12 |
| 49 | Male | 37 | LGG | Ⅱ-Ⅲ | 4 | 2 | 8 |
| 50 | Female | 32 | LGG | Ⅱ-Ⅲ | 4 | 3 | 12 |
| 51 | Female | 58 | GBM | Ⅲ | 3 | 3 | 9 |
| 52 | Male | 31 | GBM | Ⅲ | 2 | 3 | 6 |
| 53 | Male | 69 | GBM | Ⅲ | 4 | 3 | 12 |
| 54 | Female | 63 | GBM | Ⅲ | 3 | 3 | 9 |
| 55 | Female | 48 | GBM | Ⅲ | 3 | 3 | 9 |
| 56 | Female | 33 | GBM | Ⅲ | 3 | 3 | 9 |
| 57 | Male | 64 | GBM | Ⅲ | 3 | 3 | 9 |
| 58 | Male | 37 | GBM | Ⅲ | 3 | 3 | 9 |
| 59 | Male | 51 | GBM | Ⅲ | 3 | 3 | 9 |
| 60 | Female | 43 | GBM | Ⅲ | 3 | 3 | 9 |
| 61 | Male | 44 | GBM | Ⅲ | 3 | 3 | 9 |
| 62 | Male | 53 | GBM | Ⅲ | 2 | 3 | 6 |
| 63 | Female | 40 | GBM | Ⅲ | 3 | 3 | 9 |
| 64 | Male | 36 | GBM | Ⅲ | 3 | 3 | 9 |
| 65 | Male | 42 | GBM | Ⅲ | 3 | 3 | 9 |
| 66 | Female | 23 | GBM | Ⅲ | 2 | 3 | 6 |
| 67 | Female | 44 | GBM | Ⅲ | 3 | 3 | 9 |
| 68 | Male | 43 | GBM | Ⅲ | 3 | 3 | 9 |
| 69 | Male | 60 | GBM | Ⅲ | 2 | 3 | 6 |
| 70 | Male | 48 | GBM | Ⅲ | 3 | 3 | 9 |
| 71 | Female | 33 | GBM | Ⅲ-Ⅳ | 4 | 3 | 12 |
| 72 | Male | 55 | GBM | Ⅳ | 4 | 3 | 12 |
| 73 | Male | 46 | GBM | Ⅳ | 4 | 3 | 12 |
| 74 | Male | 36 | GBM | Ⅳ | 3 | 3 | 9 |
| 75 | Male | 28 | GBM | Ⅳ | 4 | 3 | 12 |
| 76 | Female | 52 | GBM | Ⅳ | 3 | 3 | 9 |
| 77 | Female | 71 | GBM | Ⅳ | 4 | 3 | 12 |
| 78 | Male | 56 | GBM | Ⅳ | 3 | 3 | 9 |
| 79 | Male | 46 | GBM | Ⅳ | 4 | 3 | 12 |
| 80 | Male | 67 | GBM | Ⅳ | 4 | 3 | 12 |
| 81 | Male | 63 | GBM | Ⅳ | 4 | 3 | 12 |
| 82 | Female | 67 | GBM | Ⅳ | 4 | 3 | 12 |
| 83 | Male | 41 | GBM | Ⅳ | 3 | 3 | 9 |
| 84 | Male | 49 | GBM | Ⅳ | 3 | 3 | 9 |
| 85 | Male | 25 | GBM | Ⅳ | 4 | 3 | 12 |
| 86 | Male | 54 | GBM | Ⅳ | 3 | 3 | 9 |
| 87 | Female | 55 | GBM | Ⅳ | 4 | 3 | 12 |
| 88 | Male | 63 | GBM | Ⅳ | 4 | 3 | 12 |
| 89 | Male | 69 | GBM | Ⅳ | 4 | 3 | 12 |
| 90 | Female | 61 | GBM | Ⅳ | 4 | 3 | 12 |
| 91 | Male | 63 | GBM | Ⅳ | 4 | 3 | 12 |
| 92 | Male | 62 | GBM | Ⅳ | 3 | 3 | 9 |
| 93 | Female | 50 | GBM | Ⅳ | 3 | 3 | 9 |
| 94 | Male | 69 | GBM | Ⅳ | 4 | 3 | 12 |
| 95 | Male | 47 | GBM | Ⅳ | 4 | 3 | 12 |
| 96 | Female | 62 | GBM | Ⅳ | 4 | 3 | 12 |
| 97 | Female | 42 | GBM | Ⅳ | 4 | 3 | 12 |
| 98 | Male | 49 | GBM | Ⅳ | 4 | 3 | 12 |
| 99 | Female | 46 | GBM | Ⅳ | 4 | 3 | 12 |
| 100 | Male | 47 | GBM | Ⅳ | 4 | 3 | 12 |
| 101 | Male | 26 | GBM | Ⅳ | 4 | 3 | 12 |
| 102 | Male | 78 | GBM | Ⅳ | 4 | 3 | 12 |
| 103 | Female | 24 | GBM | Ⅳ | 4 | 3 | 12 |
| 104 | Male | 33 | GBM | Ⅳ | 4 | 3 | 12 |
| 105 | Male | 72 | GBM | Ⅳ | 4 | 3 | 12 |
| 106 | Male | 14 | GBM | Ⅳ | 4 | 3 | 12 |
| 107 | Male | 65 | GBM | Ⅳ | 4 | 3 | 12 |
| 108 | Male | 47 | GBM | Ⅳ | 4 | 3 | 12 |
| 109 | Male | 42 | GBM | Ⅳ | 4 | 3 | 12 |
| 110 | Male | 29 | GBM | Ⅳ | 4 | 3 | 12 |
| 111 | Female | 64 | GBM | Ⅳ | 4 | 3 | 12 |
| 112 | Female | 62 | GBM | Ⅳ | 4 | 3 | 12 |
| 113 | Male | 48 | GBM | Ⅳ | 4 | 3 | 12 |
| 114 | Male | 41 | GBM | Ⅳ | 3 | 3 | 9 |
| 115 | Male | 68 | GBM | Ⅳ | 4 | 3 | 12 |
| 116 | Male | 20 | GBM | Ⅳ | 4 | 3 | 12 |
| 117 | Male | 71 | GBM | Ⅳ | 4 | 3 | 12 |
| 118 | Female | 43 | GBM | Ⅳ | 4 | 3 | 12 |
| 119 | Male | 55 | GBM | Ⅳ | 4 | 3 | 12 |
| 120 | Male | 36 | GBM | Ⅳ | 4 | 3 | 12 |
| 121 | Male | 64 | GBM | Ⅳ | 4 | 3 | 12 |
| 122 | Male | 42 | GBM | Ⅳ | 4 | 3 | 12 |
| 123 | Male | 60 | GBM | Ⅳ | 4 | 3 | 12 |
| 124 | Male | 79 | GBM | Ⅳ | 4 | 3 | 12 |
| 125 | Male | 51 | GBM | Ⅳ | 4 | 3 | 12 |

***Supplementary Table 2. Sequence of siRNA/shRNA targeting E2F7***

| **siRNA/shRNA** | **Sequence** |
| --- | --- |
| siRNA1/shRNA 1 of *E2F7* | GCAGTCTCCTGCAGGATTAAA |
| siRNA2/shRNA2 of *E2F7* | GTGCTGCCAGCCCAGATATAA |
| Universal control siRNA/shRNA | AAACGTGACACGTTCGGAGAA |

***Supplementary Table 3. Sequence of sgRNA targeting TP53***

| **sgRNA** | **Sequence** |
| --- | --- |
| sgRNA of *TP53* | CCATTGTTCAATATCGTCCG |
| Universal control sgRNA | CGCTTCCGCGGCCCGTTCAA |

**Supplementary Table 4. Primers used for qPCR**

| **Primer names** | **Sense (5’-3’)** | **Antisense (3’-5’)** |
| --- | --- | --- |
| *E2F7* | TCTGAACCCGACTGTCCCTCTT | TTTGGCAGCCACATCCAGAGTG |
| *TP53* | CCTCAGCATCTTATCCGAGTGG | TGGATGGTGGTACAGTCAGAGC |
| *ABCA2* | CATCTTGTGTGGCAACAACCG | AGGCGCGTACAGGATTTTGG |
| *ABCA3* | CTCCTCTGGAAGAACTACACCC | GGGCACATTTTCCGACTGAATC |
| *ABCA8* | TGAAATGGATGCCGATCCTTC | AGTATTGCAGTGATTTGGCCTT |
| *ABCB1* | TTGCTGCTTACATTCAGGTTTCA | AGCCTATCTCCTGTCGCATTA |
| *ABCB4* | ATAGCTCACGGATCAGGTCTC | GGATTTAGCAGCGACAAGGAAA |
| *ABCC3* | TGGGGTGAAGTTTCGTACTGG | CACGTTTGACTGAGTTGGTGATA |
| *ABCC4* | AGCTGAGAATGACGCACAGAA | ATATGGGCTGGATTACTTTGGC |
| *ABCC9* | TCAACCTGGTCCCTCATGTCT | CAGGAGAGCGAATGTAAGAATCC |
| *ABCG2* | CAGGTGGAGGCAAATCTTCGT | ACCCTGTTAATCCGTTCGTTTT |
| *GAPDH* | TGTTGCCATCAATGACCCCTT | CTCCACGACGTACTCAGCG |

**Supplementary Table 5. List of antibodies**

| **Antigen** | **Primary Antibody** | **Dilution** |
| --- | --- | --- |
| E2F7 | NOVUS; NBP1-80266; rabbit polyclonal | 1:1000 for WB |
| ABCA8 | Proteintech; 24351-1-AP; rabbit polyclonal | 1:1000 for WB |
| ABCB4 | Proteintech; 27726-1-AP; rabbit polyclonal | 1:1000 for WB |
| p53 | Abnova; MAB11254; mouse monoclonal | 1:2000 for WB |
| p-p53 (Ser46) | Cell signaling; 2521S; rabbit polyclonal | 1:1000 for WB |
| p-γH2A.X | Cell Signaling; 9718S; rabbit monoclonal | 1:1000 for WB |
| GAPDH | Proteintech; 60004-1-Ig; mouse monoclonal | 1:5000 for WB |
| β-Tubulin | Thermo Fisher Scientific; MA5-16308-1MG; mouse monoclonal | 1:2000 for WB |
| HA-tag | Abmart; M20003; mouse monoclonal | 1:5000 for WB |
| E2F7 | Proteintech; 24489-1-AP; rabbit polyclonal | 1:500 for IHC |
| p53 | Abcam; ab1101; mouse monoclonal | 5μg for ChIP |

**Supplementary Table 6. Primers used for ChIP RT-qPCR**

| **Primer name** | **Sequences (5’-3’)** | **Anti sequences (3’-5’)** |
| --- | --- | --- |
| *E2F7*-ChIP1 | TTCAATGTATGACCTAGCAGTGA | CAGGTGATCTGCCTGCCTG |
| *E2F7*-ChIP2 | ACACTCTAGCCTGGGTGACA | ACTGAAATGTGAGACAGCCTTCT |
| *E2F7*-ChIP2 | TTTCTAGACTGTCTCCCAGAATGC | GCCCTGGAGACAAGACTGGA |
| *E2F7*-ChIP4 | ACACCAGCGTGCGATGTTA | TGGAAGAACGATCTACACCCTC |
